# Effects of unilateral neck muscle vibration on tilt direction and variability of subjective postural vertical in the frontal plane during seated posture in healthy adults

**DOI:** 10.7717/peerj.20579

**Published:** 2026-01-07

**Authors:** Yuji Fujino, Kazu Amimoto, Tadamitsu Matsuda, Daisuke Sekine, Toshiyuki Fujiwara

**Affiliations:** 1 Department of Physical Therapy, Faculty of Health Science, Juntendo University, Bunkyo-Ku, Tokyo, Japan; 2 Department of Rehabilitation Science, Faculty of Rehabilitation Science, Sendai Seiyo Gakuin University, Sendai, Miyagi, Japan; 3 Department of Rehabilitation Center, Saitama Medical University International Medical Center, Hidaka, Saitama, Japan; 4 Department of Rehabilitation Medicine, Juntendo University Graduate School of Medicine, Bunkyo-ku, Tokyo, Japan

**Keywords:** Proprioception, Vibration, Posture, Perception, Spatial orientation, Postural balance

## Abstract

**Background:**

Unilateral neck muscle vibration (NMV) activates the primary endings of muscle spindles and modulates both subjective visual vertical and subjective straight-ahead perception. However, its effects on subjective postural vertical (SPV), crucial for postural balance, remain poorly understood. We aimed to investigate the effects of unilateral NMV-induced proprioceptive stimulation on SPV tilt direction and intraindividual variability in the frontal plane in healthy participants.

**Methods:**

We included 48 healthy adults (29 males, 19 females; age 22.5 ± 1.1 years; height 167.7 ± 7.4 cm; weight 58.7 ± 8.4 kg), randomly divided into four groups: vibrations to the left (L-Vib) and right sides (R-Vib), as well as sham stimulations to the left (L-Sham) and right (R-Sham). Vibration was applied for 10 min at 80 Hz with an amplitude of 0.8 mm. SPV was measured using a motorized vertical-tilting chair equipped with a backrest and lateral supports. Participants were seated without ground contact, with their trunk fixed and arms crossed; the ir head and legs re mained unrestrained. The experimenter tilted the chair from an initial position of 15° or 20° in the frontal plane toward the vertical at a speed of 1.5°/s. A digital inclinometer recorded the tilt angle when participants reported their body felt upright. Each session comprised eight trials with pseudorandom starting directions and angles. The mean tilt direction and standard deviation across trials were calculated. SPV was assessed before, during, and after stimulation. A two-way analysis of variance was conducted to analyze the effect s of unilateral NMV on SPV outcomes.

**Results:**

There were no significant demographic differences across groups. For SPV tilt direction, there was no statistically significant interaction between group and time. However, for SPV variability, significant effects were observed for time (F1,44 = 9.591, *p* = 0.003, partial η^2^ = 0.179) and the interaction between group and time (F6,44 = 2.325, *p* = 0.039, partial η^2^ = 0.137). Participants in the L-Vib group exhibited significantly reduced variability both during and after stimulation compared with those in the L-Sham (*p* = 0.004) and R-Sham (*p* < 0.001) groups. Similarly, participants in the R-Vib group showed significantly lower variability than those in the R-Sham group (*p* = 0.02).

**Discussion:**

These findings highlight the role of sensorimotor integration in body orientation and suggest that unilateral NMV may enhance the precision of verticality estimation. Based on this preliminary study, NMV could be a promising intervention for individuals with SPV abnormalities.

## Introduction

The concept of an “internal representation” of verticality, integrated and updated by information from vestibular, somatosensory, and visual sources (Barra et al., 2010), is a theoretical framework widely used in behavioral neuroscience. However, this notion remains debated. Alternative models have been proposed, including the equilibrium-point hypothesis (Feldman, 2015), which posits that postural perception and control emerge from interactions between neural commands and biomechanical constraints. This internal representation can be altered by neurological disorders, particularly vestibular disease or stroke. Sensory stimulation is known to influence posture by modulating one or more sources of input (Bonan et al., 2015). Specifically, unilateral neck muscle vibration (NMV) has been shown to influence postural orientation and spatial perception, particularly through its effects on proprioceptive inputs (Pettorossi & Schieppati, 2014; Jamal et al., 2020).

NMV is a potent stimulus that activates various types of sensory afferents, including Ia and II muscle spindle fibers, as well as cutaneous receptors (Burke et al., 1976a, 1976b; Roll & Vedel, 1982; Fallon & Macefield, 2007; Lowrey, Strzalkowski & Bent, 2013; Strzalkowski, Ali & Bent, 2017), thereby modulating proprioceptive signaling. Thus, it would be oversimplified to state that NMV selectively activates only primary endings of muscle spindles. The proprioceptive receptors in cervical muscles, including muscle spindles and Golgi tendon organs, play a key role in body orientation perception and detecting the head’s position relative to the trunk (Pettorossi & Schieppati, 2014). However, the direct anatomical connection between cervical proprioceptors and vestibular or oculomotor pathways has not clearly been established (Kavounoudias et al., 1999).

Spatial orientation is often evaluated using perceptual measures, including subjective visual vertical (based on visual input), subjective straight-ahead (based on perceived forward direction), and subjective postural vertical (SPV; based primarily on vestibular and proprioceptive cues).

Previous studies have shown that unilateral NMV influences the perception of vertical and straight-ahead orientation: the subjective visual vertical in the frontal plane and the subjective straight-ahead in the horizontal plane both tilt toward the stimulated side (Karnath et al., 2002; McKenna, Peng & Zee, 2004; Ceyte et al., 2006; Fraser, Makooie & Harris, 2015). Conversely, unilateral NMV tilts the center of foot pressure in the frontal plane toward the contralateral side, influencing body orientation (Kavounoudias et al., 1999; Courtine et al., 2007). The neck muscles, along with visual and vestibular inputs, are integral to the perception of body orientation in space (Biguer et al., 1988). However, the effect of NMV on SPV, a key measure of body orientation, remains unclear.

We hypothesized that unilateral NMV would modulate SPV in healthy adults, particularly by altering tilt direction and reducing variability through proprioceptive input, independent of visual information. Given that verticality perception deficits have been implicated in postural disorders, such as in stroke or Parkinson’s disease, it is necessary to first clarify how NMV affects SPV in a controlled, non-pathological context. Notably, the temporal profile of NMV-induced effects, whether they occur during stimulation, persist afterward, or both, has not been systematically characterized. In this study, we investigated the immediate and short-term effects of unilateral NMV on SPV direction and variability in healthy adults. By clarifying these effects and their timing, we seek to contribute to the neurophysiological understanding of verticality perception and to establish foundational knowledge for future translational applications.

## Materials and Methods

### Participants

Healthy young individuals with no history of neurological, musculoskeletal, or psychiatric disorders were recruited from Juntendo University. Participants were recruited through public advertisements targeting undergraduate and graduate students at Juntendo University, aged 20 to 39 years. The SPV of the frontal plane was assessed in 48 right-handed healthy adults (29 males, 19 females; age 22.5 ± 1.1 years; height 167.7 ± 7.4 cm; weight 58.7 ± 8.4 kg). Only right-handed individuals were included to control for potential lateralization effects in spatial perception, as hemispheric dominance may influence verticality judgments. Exclusion criteria included: difficulty understanding the procedures; presence of vestibular symptoms such as dizziness; neurological, psychiatric, or orthopedic conditions that could interfere with postural assessment; and inability or unwillingness to provide written informed consent. Participants were randomly divided into four groups for the analysis of unilateral NMV effects: vibration to the left side (L-Vib), vibration to the right side (R-Vib), sham stimulation to the left (L-Sham), and sham stimulation to the right (R-Sham). The group assignment was performed using computer-generated block randomization with a block size of four to ensure equal distribution across the four groups.

Outcome assessments and data analysis were primarily conducted by a single assessor (YF), who was aware of group assignments. However, since all measurements were obtained using an automated tilt device and analyzed based on predefined criteria, the risk of evaluator bias was considered minimal.

**Figure 1:**
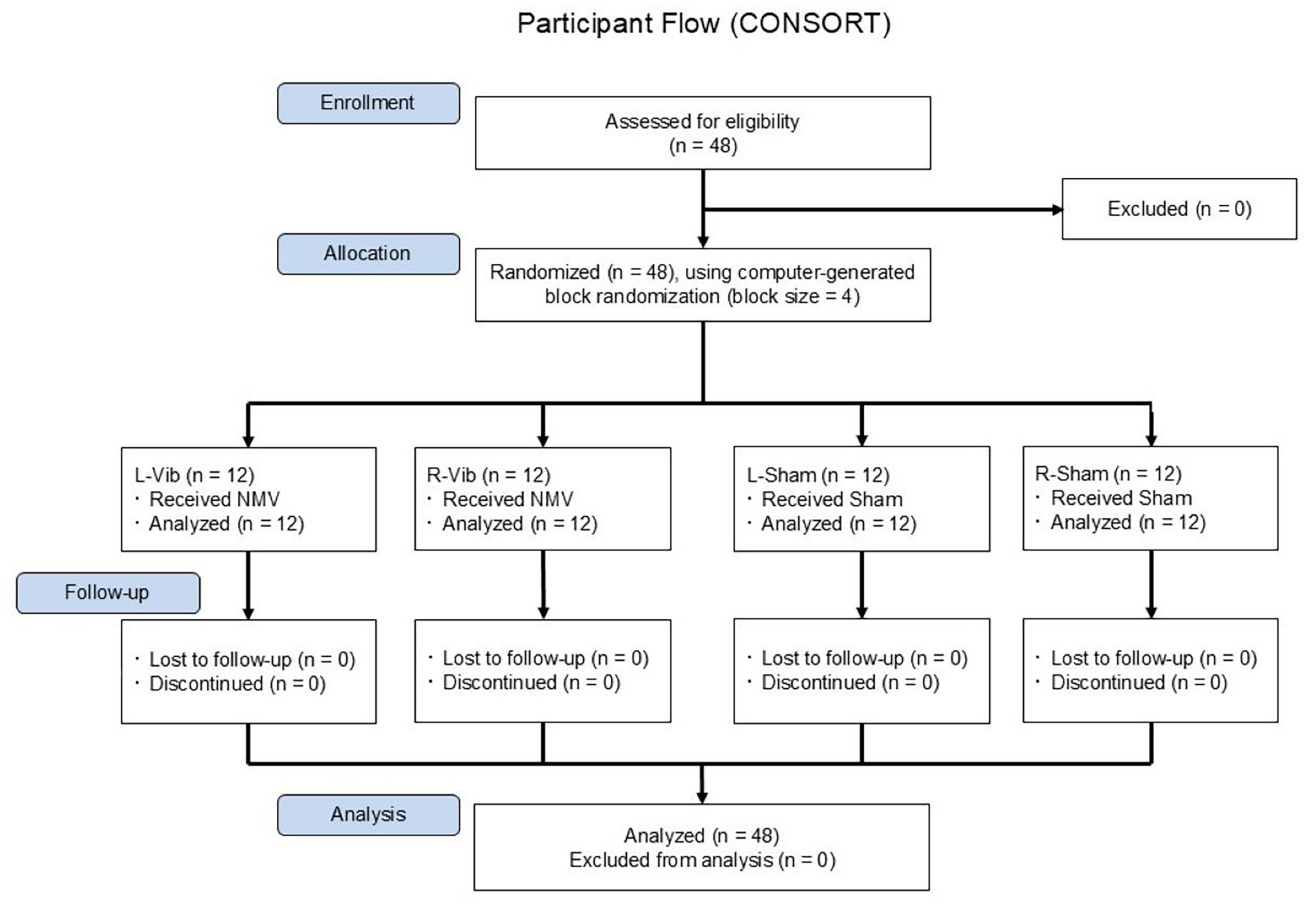
CONSORT-style flowchart of participant enrollment, randomization, and assessment timeline. A total of 48 healthy right-handed adults were assessed for eligibility, enrolled, and randomly assigned using computer-generated block randomization (block size = 4) to one of four groups: L-Vib, R-Vib, L-Sham, or R-Sham. All participants completed the intervention and were included in the final analysis. No dropouts occurred. NMV, neck muscle vibration; L-Vib, vibration to the left side; R-Vib, vibration to the right side; R-Sham, sham stimulation to the right side; L-Sham, sham stimulation to the left side. Note: No long-term follow-up was conducted in this study. “Follow-up” here refers to the immediate post-intervention period, during which all participants completed all scheduled assessments.

The randomization sequence was generated by an investigator not involved in recruitment or assessment, and group allocation was concealed using sealed, opaque envelopes until after participant enrollment. Figure 1 illustrates the overall study flow, including recruitment, group allocation, and measurement timeline. All participants provided written informed consent prior to participation. The study was approved by the institutional review board of Juntendo University (approval number: 21-007) on June 29, 2021. We employed a single-blind, between-subjects, randomized controlled design in this study. All randomized participants completed the experimental protocol and were included in the final analysis. No dropouts or missing data occurred during the study period.

*A priori* power analysis was conducted using G*Power 3.1 (Faul et al., 2009) to determine the minimum required sample size for detecting a medium effect size (f = 0.25) in a two-way repeated-measures analysis of variance (ANOVA). This analysis utilized an alpha level of 0.05 and a desired power of 0.80. The chosen effect size corresponds to the conventional classification for a medium effect, as defined by Cohen (1988). The analysis indicated that a minimum of 24 participants would be required. Our final sample size of 48 exceeded this threshold, ensuring adequate statistical power for detecting meaningful effects.

This study was registered with the University Hospital Medical Information Network Clinical Trials Registry (UMIN-CTR, ID: UMIN000039747) prior to enrollment. The registered protocol outlines a broader research framework, and the present report focuses on a specific subset involving the effects of unilateral NMV on SPV. The registry describes the study as open-label; however, a single-blind design was implemented, in which participants were unaware of their group allocation. Although participants could perceive somatosensory feedback from the vibration (*e.g*., skin or muscle sensation), the stimulation device emitted no noticeable sounds or visible indicators during operation. Participants were also instructed to keep their eyes closed throughout the procedure, effectively preventing them from visually identifying whether the stimulation was active. Therefore, in our opinion, the blinding was sufficient to ensure that participants remained unaware of their group assignment. The intervention protocols, grouping, and primary outcomes reported here are consistent with the registered objectives, with additional procedural detail provided to enhance scientific transparency.

### Subjective postural vertical measurement

**Figure 2:**
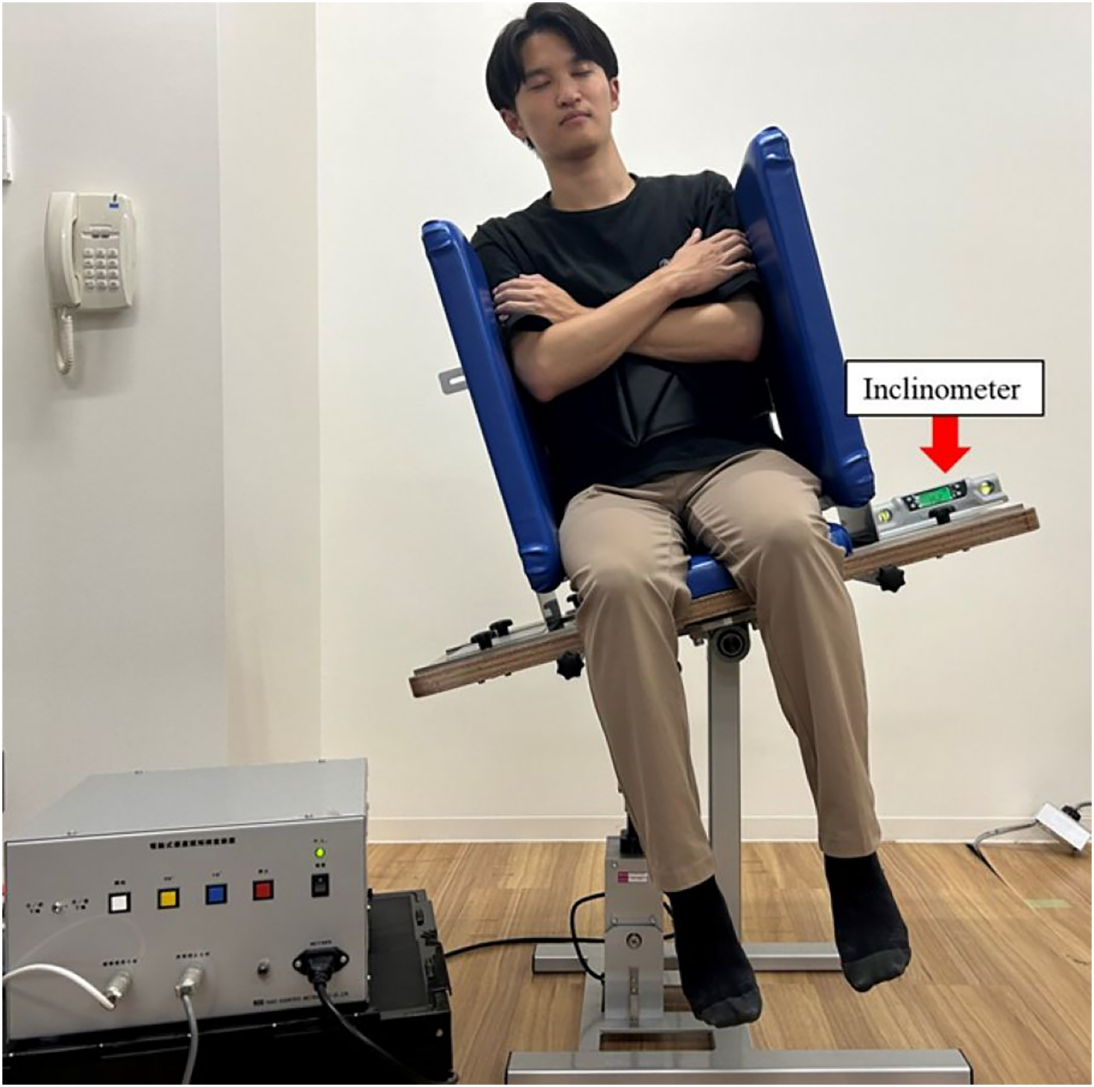
Assessment setup for measuring subjective postural vertical using a motorized vertical-tilting chair. The experimenter tilted the seat from an initial angle of 15° or 20° toward the vertical in the frontal plane at a constant speed of 1.5°/s. A digital inclinometer was affixed to the lateral surface of the seat base, which moved with the participant, allowing measurement of whole-body tilt. Participants sat without ground contact, with their trunk secured to a backrest, arms crossed, and their head and legs unrestrained. Visual input was eliminated by having participants keep their eyes closed. Photo credit: Yuji Fujino.

SPV was measured using a motorized vertical-tilting chair (hereafter referred to as the “tilting chair”; Takei Scientific Instruments Co., Ltd., Niigata, Japan) (Fig. 2), a motor-driven device capable of setting a specific inclination speed. The lateral wall width was adjusted to fit each participant’s body. Participants sat on the tilting chair without ground contact, with their trunk fixed and arms crossed over their chest. Their heads and legs were unrestrained, and their eyes were closed. No specific instructions were provided regarding head position. This approach was chosen to allow participants to maintain a natural head position relative to their fixed trunk, thereby approximating habitual head–trunk alignment in seated posture. SPV was measured using the experimenter adjustment method (Fukata et al., 2019), where the experimenter operated the tilting chair. The seat tilt angle was measured using a digital inclinometer (Myzox Co., Ltd., Digilevel Compact, Aichi, Japan) affixed to the lateral side of the seat base, which tilted together with the participant, enabling the quantification of whole-body tilt in the frontal plane. A schematic of the setup is illustrated in Fig. 2. The experimenter tilted the vertical-tilting chair seat from an initial angle of either 15° or 20° in the frontal plane at a speed of 1.5°/s until participants verbally indicated they had reached a true vertical position, at which point the angle was recorded. Eight trials were conducted in a balanced pseudorandom sequence, such as ABBABAAB, where A and B denoted the tilt direction (left or right) rather than the tilt angle. Starting angles (15° and 20°) were evenly distributed within each direction across trials for each participant, and four counterbalanced trial patterns were used across participants to minimize order effects. A true vertical position was defined as 0°, with rightward and leftward tilts recorded as positive and negative, respectively. The mean tilt direction and standard deviation (SD, variability) of the eight trials were calculated. Tilt direction was defined as the magnitude and direction of vertical perception, while variability was defined as intraindividual variability, calculated as the SD of the eight SPV angles recorded per session for each participant. For each trial, we also recorded the starting angle (15° or 20°) and the initial inclination side, defined as the side from which the chair started to return toward the vertical alignment (left or right). These trial-level labels were counterbalanced so that starting angles were evenly distributed for each initial side and time point (before, during, after).

### Vibration stimulation and procedures

Vibration was delivered using a device developed by Ito et al. (2020) (Aim Co., Ltd., Aichi, Japan), comprising an amplifier and a circular tactile transducer. The transducer was positioned approximately 3–4 cm below the external occipital protuberance and slightly lateral to the midline, corresponding anatomically to the region over the superficial portions of the splenius capitis and semispinalis capitis. However, because cervical muscles are densely layered, deeper fibers, such as the multifidus, were likely co-stimulated. Consistent with previous studies in which the location was described broadly as “posterior/dorsal neck muscles” (Karnath et al., 2002; McKenna, Peng & Zee, 2004), we referred to the stimulated region collectively as the posterior cervical muscle group while acknowledging that selective activation of a single muscle cannot be guaranteed. This location was chosen to provide laterally biased proprioceptive input relevant to postural control, consistent with previous studies (Pettorossi & Schieppati, 2014; Jamal et al., 2020). The transducer (35 mm diameter) operated at a fixed amplitude of 0.8 mm and a frequency of 80 Hz. Although the controller allowed adjustments from 70 to 120 Hz, frequency of 80 Hz was selected for this study because this frequency lies within the range known to robustly drive muscle spindle primary endings (70–100 Hz) based on microneurography (Roll & Vedel, 1982; Fallon & Macefield, 2007). Furthermore, the 0.8 mm amplitude follows common practice in behavioral NMV protocols and device specifications (Jamal et al., 2020).

The transducer was affixed to the skin using surgical tape applied in a cross (+) configuration, with tape orientation adjusted as needed to avoid adhering to hair; additionally, an elastic strap was used for secure placement. Participants maintained a stable posture without significant head movement throughout the procedure, and no displacement or detachment of the device occurred during the trials.

During the vibration condition, SPV was measured beginning at the 5-min mark of the 10-min stimulation period and continued until completion of all eight trials (within approximately 5 min), allowing assessment during the active vibratory input. Post-assessment (“after”) began 5 min after stimulation offset. These time intervals were standardized for all participants.

All SPV measurements were conducted by a trained assessor (YF) who was unblinded to group assignment (vibration *vs*. sham) and the side of stimulation. To reduce measurement bias, tilt angles were captured with a digital inclinometer, and the SPV protocol—timing, starting angles, and trial order—was standardized for all participants. This was a single-blind evaluation. Participants were blinded to allocation, and their eyes remained closed throughout the experimental period to eliminate visual cues.

The stimulation duration (10 min) was chosen to balance efficacy and feasibility, consistent with previously described neck-vibration protocols that reported robust perceptual/postural effects with multi-minute stimulation (*e.g*., Karnath et al., 2002; Jamal et al., 2020) and were supported by pilot testing for tolerability. The number of repetitions (eight SPV trials per session) was adopted from previous studies using similar experimental paradigms for subjective verticality measures (Fukata et al., 2019), ensuring a balance between statistical reliability and participant burden.

**Figure 3:**
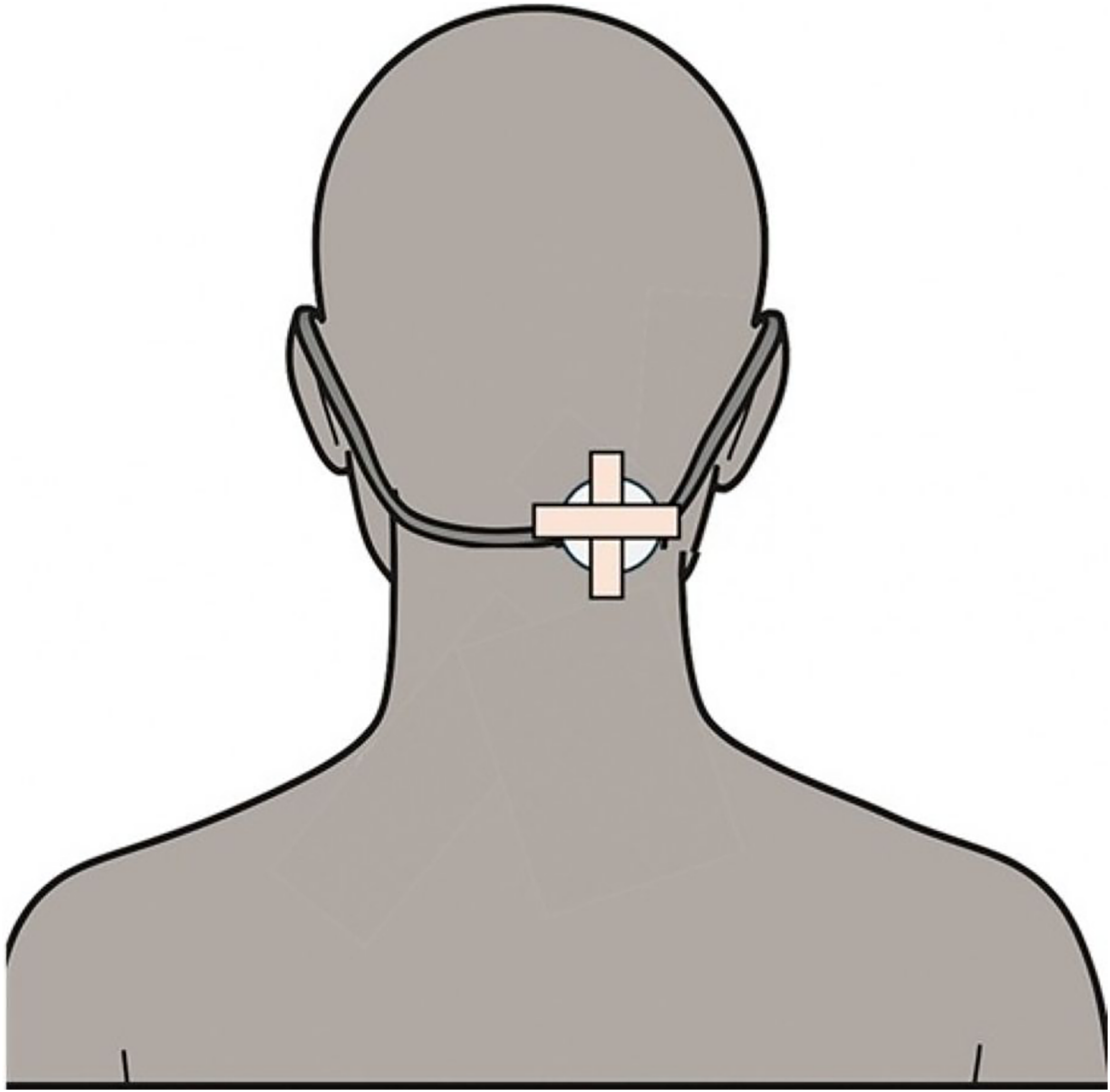
Placement and fixation of the vibration device. The vibrator was affixed approximately 3–4 cm below the external occipital protuberance and slightly lateral to the midline, corresponding to the posterior cervical muscle region. The device was secured using surgical tape and elastic straps to ensure stability during stimulation. This location was selected to target proprioceptive input relevant to verticality perception, consistent with prior studies.

In the sham condition, the same transducer was attached in the same position with identical fixation, but the device remained powered off, delivering no vibration and ensuring a comparable tactile sensation and appearance without delivering actual proprioceptive input. Participants received NMV or sham stimulation on either the left or right side for 10 min. SPV was assessed at three time points relative to the stimulation protocol: immediately before stimulation (“before”), beginning precisely 5 min after stimulation onset (“during”), and beginning 5 min after stimulation cessation (“after”). Each SPV measurement session comprised eight trials and was completed within approximately 5 min. Figure 3 illustrates the vibration stimulation setup, including the placement and fixation method of the transducer.

### Statistical analysis

The demographic data of the four groups were compared using one-way ANOVA for continuous variables and the chi-squared test for categorical variables. A two-way repeated-measures ANOVA was employed to compare changes in SPV between groups at the three time points. Group and time were used as factors to assess the presence of interactions. When a significant interaction was identified, a simple main effects test was performed. *Post hoc* pairwise comparisons were conducted using Bonferroni-adjusted tests to control for multiple comparisons.

Exploratory supplementary analyses evaluated potential influences of trial-level factors (starting angle, initial side). At the trial level we fitted a linear mixed-effects model with participant-specific random intercepts and fixed effects for group (L-Vib, R-Vib, L-Sham, R-Sham), time point (before, during, after), starting angle (15° *vs*. 20°), initial side (left *vs*. right), and the interactions time point × starting angle and time point × initial side. The dependent variable was the SPV angle in degrees (rightward positive). Furthermore, to evaluate intra-individual variability, we computed the within-participant SD for each time point × starting angle × initial side cell (two trials per cell) and analyzed these SDs with participant fixed effects and cluster-robust standard errors. These supplementary models complement the primary ANOVA framework and do not alter it.

Prior to performing ANOVAs, we assessed the normality of the SPV data using the Shapiro–Wilk test. No substantial deviations from normality were observed, supporting the use of parametric methods.

Homogeneity of variance was assessed using Levene’s test, and no significant violations were found across all time points and conditions (all *p* > 0.05). Sphericity was tested using Mauchly’s test and was not violated for either SPV variability or tilt direction (*p* = 0.581 and *p* = 0.547, respectively). When appropriate, Greenhouse–Geisser corrections were prepared to be applied.

Statistical analyses were conducted using SPSS Statistics version 27.0 (IBM Corp, Tokyo, Japan), with significance set at *p* < 0.05.

## Results

The demographic data of the four groups are presented in Table 1. No significant differences were found in age (F3,44 = 1.617, *p* = 0.199), sex (χ^2^(3) = 1.778, *p* = 0.620), weight (F3,44 = 0.801, *p* = 0.500), or height (F3,44 = 0.900, *p* = 0.462) across the four groups. No participants reported any discomfort, dizziness, or adverse effects during or after the NMV procedure.

**Table 1:** Demographic data of four groups.

	L-Vib (*n* = 12)	L-sham (*n* = 12)	R-Vib (*n* = 12)	R-sham (*n* = 12)	*p*
Age (years)	22.8 ± 1.5	22.3 ± 0.8	22.9 ± 1.1	22.1 ± 0.8	0.199^a^
Sex (male/female)	8 (66.7)/4 (33.3)	6 (50.0)/6 (50.0)	7 (58.3)/5 (41.7)	9 (75.0)/3 (25.0)	0.620^b^
Weight (kg)	60.2 ± 6.1	55.7 ± 8.4	58.7 ± 6.8	60.5 ± 11.7	0.500^a^
Height (cm)	169.6 ± 5.9	165.6 ± 8.9	166.3 ± 6.6	169.2 ± 8.1	0.449^a^

**Note:**

Continuous data are presented as mean ± standard deviation, while categorical data are presented as n (%). L, Left side; R, Right side; Vib, Vibration.

a One-way analysis of variance.

b Chi-square test.

**Figure 4:**
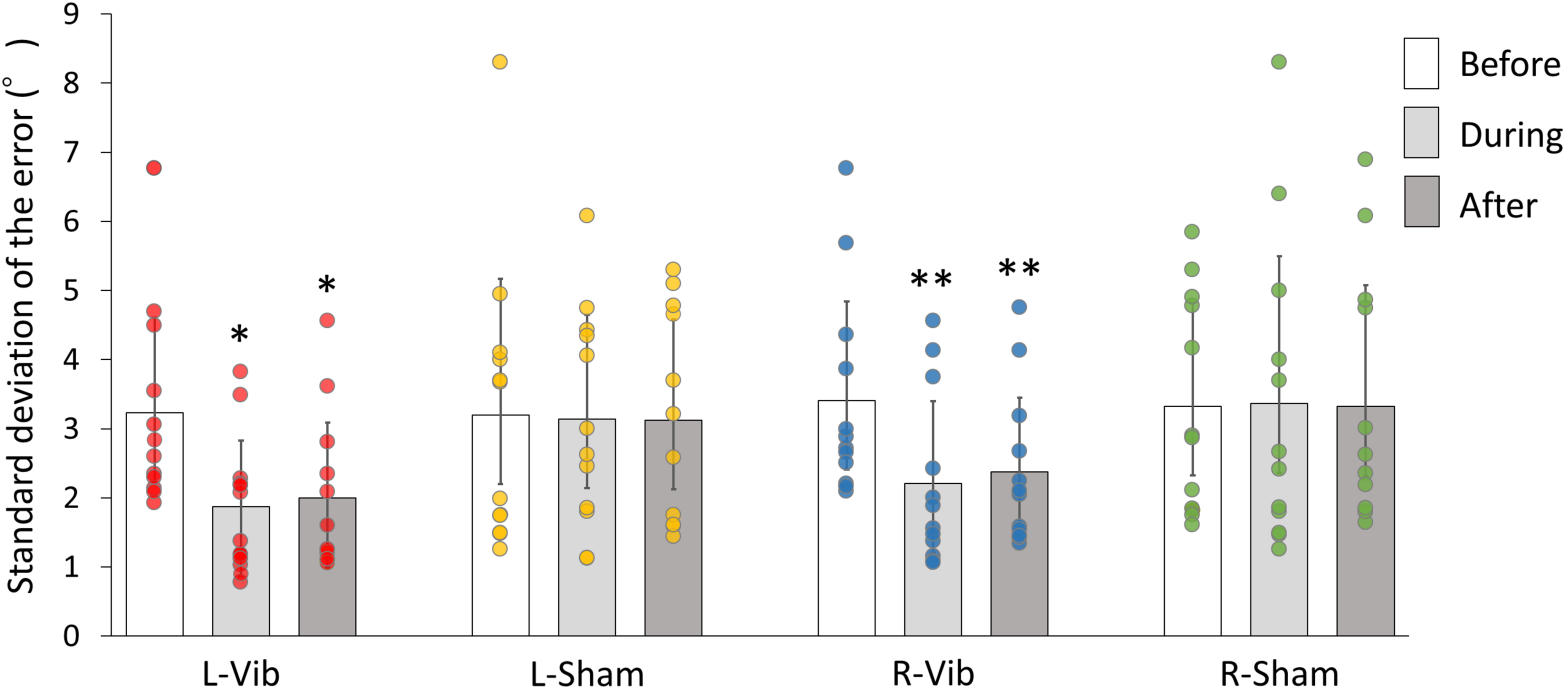
Bar graphs and scatter plots depicting the simple main effects on SPV variability. Bar graphs indicate the value of variability and scatter plots show individual data points. An asterisk (*) means that L-Vib group exhibited significantly less variability both during and after stimulation compared with that of the L-sham and R-sham groups. Two asterisks (**) mean that the R-Vib group showed significantly less variability both during and after stimulation compared with that of the R-sham group. SPV, subjective postural vertical; L-Vib, vibration to the left side; R-Vib, vibration to the right side; R-Sham, sham stimulation to the right side; L-Sham, sham stimulation to the left side.

The SPV measurement results are shown in Table 2. For the tilt direction of SPV, there was no significant interaction between group and time (F6,44 = 0.629, *p* = 0.707, partial η^2^ = 0.041). However, for SPV variability, significant differences were observed for time (F1,44 = 9.591, *p* = 0.003, partial η^2^ = 0.179), and for the interaction between group and time (F6,44 = 2.325, *p* = 0.039, partial η^2^ = 0.137). Table 3 shows the detailed means and SDs for SPV variability across sessions and groups. In the analysis of simple main effects on SPV variability (Fig. 4), the participants in the L-Vib group exhibited significantly less variability both during and after stimulation compared with those in the L-sham (*p* = 0.004) and the R-sham (*p* < 0.001) groups. Similarly, the participants in the R-Vib group showed significantly less variability compared with those in the R-sham group (*p* = 0.02). In absolute terms, the L-Vib group exhibited an average reduction in SPV variability of about 1.2° from before to during (Table 3) time points, indicating a sizable narrowing of trial-to-trial dispersion that was not explained by starting angle or initial side in the exploratory models.

**Table 2:** Effects of neck muscle vibration on subjective postural vertical tilt direction (°) across sessions.

Tilt direction (°)				
Session	L-Vib	L-sham	R-Vib	R-sham
Before	−0.2 ± 1.0	0.1 ± 0.7	−0.3 ± 0.7	−0.2 ± 0.7
	95% CI [−0.84 to 0.44]	95% CI [−0.34 to 0.54]	95% CI [−0.74 to 0.14]	95% CI [−0.64 to 0.24]
During	−0.2 ± 0.7	0.1 ± 0.9	−0.2 ± 0.7	−0.1 ± 0.8
	95% CI [−0.64 to 0.24]	95% CI [−0.47 to 0.67]	95% CI [−0.64 to 0.24]	95% CI [−0.61 to 0.41]
After	−0.2 ± 0.6	−0.1 ± 0.7	−0.2 ± 0.6	−0.5 ± 0.8
	95% CI [−0.58 to 0.18]	95% CI [−0.54 to 0.34]	95% CI [−0.58 to 0.18]	95% CI [−1.01 to 0.01]
**Effect**	**F (df)**	***p*-value**	**Effect size (partial η^2^)**	
Group	F (3, 44) = 0.615	0.609	0.040	
Time	F (1, 44) = 0.622	0.435	0.014	
Group × Time	F (6, 44) = 0.629	0.707	0.041	

**Notes:**

Continuous data are presented as mean ± standard deviation. CI, 95% confidence interval; L, Left side; R, Right side; Vib, Vibration. Partial η^2^ = partial eta squared, indicating effect size.

**Table 3:** Effects of neck muscle vibration on subjective postural vertical variability (°) across sessions.

Variability (°)				
Session	L-Vib	L-sham	R-Vib	R-sham
Before	3.2 ± 1.4	3.2 ± 2.0	3.4 ± 1.4	3.3 ± 1.5
	95% CI [2.31–4.09]	95% CI [1.93–4.47]	95% CI [2.51–4.29]	95% CI [2.35–4.25]
During	1.9 ± 1.0	3.1 ± 1.5	2.2 ± 1.2	3.4 ± 2.1
	95% CI [1.26–2.54]	95% CI [2.15–4.05]	95% CI [1.44–2.96]	95% CI [2.07–4.73]
After	2.0 ± 1.1	3.1 ± 1.5	2.4 ± 1.1	3.3 ± 1.8
	95% CI [1.30–2.70]	95% CI [2.15–4.05]	95% CI [1.70–3.10]	95% CI [2.16–4.44]
**Effect**	**F (df)**	***p*-value**	**Effect size (partial η^2^)**	
Group	F (3, 44) = 1.258	0.301	0.079	
Time	F (1, 44) = 9.591	0.003*	0.179	
Group × Time	F (6, 44) = 2.325	0.039*	0.137	

**Notes:**

Continuous data are presented as mean ± standard deviation. CI, 95% confidence interval; L, Lest side; R, Right side; Vib, Vibration.

* Statistically significant difference (*p* < 0.05). Partial η^2^ = partial eta squared, indicating effect size.

Additionally, we fitted trial-level models that included starting angle (15° *vs*. 20°) and initial side (left *vs*. right) and their interactions with time point. For SPV angle, neither starting angle (*p* = 0.309) nor initial side (*p* = 0.996) showed a main effect, and interactions with time point were not significant (time point × starting angle: *p* = 0.410 [during], 0.386 [after]; time point × initial side: *p* = 0.642 [during], 0.719 [after]). For intra-individual variability (cell-wise SD per time point × starting angle × initial side), neither starting angle (*p* = 0.340) nor initial side (*p* = 0.215) was significant. A small time point × initial side effect at the ‘after’ time point reached *p* = 0.049 but did not persist after adjusting for multiple testing. The group × time point pattern for variability remained materially unchanged when these factors were included.

## Discussion

In this study, we investigated the influence of unilateral NMV on SPV, a critical factor for maintaining balance. Our results showed that unilateral NMV improved the precision of SPV responses during stimulation, regardless of the side of the stimulus, as reflected by reduced variability but unchanged tilt direction. Additionally, an aftereffect was observed with left-sided NMV, suggesting that unilateral NMV may continue to affect balance function post-stimulation.

Unilateral NMV is known to shift the subjective visual vertical in the frontal plane and the subjective straight-ahead in the horizontal plane toward the stimulated side (Karnath et al., 2002; McKenna, Peng & Zee, 2004; Ceyte et al., 2006; Fraser, Makooie & Harris, 2015), while postural orientation shifts to the contralateral side (Kavounoudias et al., 1999; Courtine et al., 2007). We hypothesized that SPV would shift to the contralateral side of the stimulation, similar to postural orientation; however, no significant change in SPV tilt direction was found. This may be attributed to the specific testing conditions, including immobilization of the trunk, absence of visual input due to eye closure, and the passive nature of the tilt task, all of which likely limited sensorimotor adaptation and spatial recalibration processes. However, other sensory cues, including skin contact, changes in pressure distribution, and mechanical tension in lower limb joints, may still have contributed to the postural estimate even in the absence of visual input and should be considered in future investigations. Additionally, because this was a single-blind study carried out by an unblinded assessor, residual measurement bias cannot be fully excluded despite the use of a digital inclinometer and a standardized protocol. To examine potential dependencies in starting conditions, we conducted exploratory trial-level analyses including starting angle (15° *vs*. 20°) and initial inclination side (left *vs*. right) and their interactions with time point; none of the studied factors showed a systematic effect on SPV variability, nor did the interactions account for the during-session reduction. Thus, the observed decrease in variability is unlikely to reflect an artifact tied to the starting conditions.

Beyond measurement conditions, the lack of directional SPV shift may also reflect the nature of sensory reintegration required for updating internal spatial representations. Unlike subjective visual vertical or active postural tasks, SPV assessment under passive tilting and restricted movement conditions may suppress the dynamic multisensory recalibration that typically occurs in more ecologically valid or motion-rich settings. This notion aligns with previous findings suggesting that proprioceptive stimulation alone may be insufficient to alter perceived verticality when integration with visual or motor cues is limited (Barra et al., 2010; Leplaideur et al., 2015). Thus, while NMV can modulate vertical perception variability, its effect on directional bias may depend on the degree of sensorimotor engagement and the availability of conflicting or reinforcing spatial cues.

Importantly, the observed reduction in SPV variability suggests that unilateral NMV may enhance the consistency of internal estimates of verticality. This may be mediated by the increased reliability of proprioceptive input from neck muscles, resulting from the selective activation of Ia afferents, leading to more stable integration within sensorimotor pathways involved in postural orientation. The mean tilt direction remained unchanged; however, the reduction in variability reflects an enhancement of perceptual precision rather than accuracy. This distinction is important because elevated SPV variability has been reported in individuals with stroke or vestibular disorders.

The observed effect size for the interaction between group and time on SPV variability (partial η^2^ = 0.137) represents a large effect, suggesting that the consistency of verticality perception was meaningfully enhanced. Previous clinical reports have linked increased SPV variability with lateropulsion and postural instability, especially in stroke survivors (Fukata et al., 2020). Therefore, reducing SPV variability may lead to improved postural stability and spatial orientation.

Further, the magnitude of the change was meaningful beyond statistical significance. A reduction of about 1.2° from the before to during stimulation time points in the L-Vib group (Table 3) represents a clear tightening of the within-session response spread. While direct numerical comparisons with the stroke cohorts reported by Fukata et al. (2020) are limited by methodological and population differences, a reduction of this order counters the pathological increases in variability associated with lateropulsion and may hold translational relevance pending confirmation through future studies.

Our findings suggest that unilateral NMV may serve as a neuromodulatory intervention to reinforce somatosensory precision and improve postural control in these populations. While full sensory isolation was not achieved, as other bodily cues such as pressure distribution and skin stretch could still inform spatial perception, the observed reduction in SPV variability suggests a stabilizing effect of NMV. This may reflect the capacity of proprioceptive stimulation to enhance perceptual consistency even under simplified experimental conditions. NMV is thought to persistently modify vestibular-derived self-motion perception and interact with the adaptive processes of the vestibular system, potentially leading to the consolidation of a new spatial coordinate system (St George, Day & Fitzpatrick, 2011). Moreover, in determining the direction of gravity, verticality is estimated by the head and body separately, and the bimodal SPV formed by the mutual complementation and integration of sensorimotor information from both is converted *via* neck proprioception (Fraser, Makooie & Harris, 2015). Cervical proprioceptor activation is believed to influence spatial references, motion perception, and locomotor orientation, adapting perceptive responses to motor biases and novel postural settings (Pettorossi & Schieppati, 2014). Based on these findings, we suggest that the proprioceptive receptors of the cervical muscles may contribute to the precision of postural vertical perception, even in conditions where body geometry remains unchanged.

The persistence of effects after unilateral NMV cessation is believed to result from the activation of proprioceptive receptors in the cervical muscles and the plastic integration of sensory information in the central network (Grassi & Pettorossi, 2001; Wolpaw & Tennissen, 2001; Lynch, 2004; Straka et al., 2005). The duration of unilateral NMV effects on postural orientation has been shown to range from 3 min to 3 h, though individual differences have been shown (Wierzbicka, Gilhodes & Roll, 1998; Duclos et al., 2007; Leplaideur et al., 2015). The aftereffects observed 10 min post-stimulation in this study fall within the reported time range, suggesting consistency with previous findings. Individual variability in aftereffects may also explain the differences in significance between the NMV and sham conditions for right- and left-sided stimuli.

In this study, the participants’ heads were not fixed to allow attachment of the vibration transducer. The coincidence of vestibular and somatosensory gravity perception has been shown to be important for stable judgments of postural verticality (Barra et al., 2010). While participants did not voluntarily move their necks, the possibility remains that this may have influenced posture and measurement data across participants. Although the current study employed a between-subjects design with four distinct groups, a within-subject repeated-measures design might have provided stronger control over inter-individual variability and increased statistical power. This design choice was made to avoid potential carryover effects and because implementing repeated NMV sessions with sufficient washout periods was impractical. Nonetheless, future studies should consider using a within-subject crossover design to more robustly examine the effects of NMV on SPV. Furthermore, because of the limited sample size of this study, the generalizability of the results should be cautiously considered when applying them to other populations. Nevertheless, this is the first report in which the effects of unilateral NMV on SPV in the frontal plane were examined. Previous studies have shown that unilateral NMV can shift the subjective visual vertical and the subjective axis of posture in the direction opposite to the stimulus (*e.g*., Karnath et al., 2002; Courtine et al., 2007); however, our findings showed no shift in tilt direction, although a significant reduction in SPV variability was observed. Given that abnormalities in SPV variability have also been associated with postural disorders in patients with stroke (Fukata et al., 2020), future research should investigate the potential therapeutic application of NMV in relevant patient populations, especially those with pushing behavior with increased SPV instability, while considering its effects on SPV in healthy individuals.

Furthermore, the substantial inter-individual variability observed in SPV responses highlights the need for future studies aimed at investigating the contributing factors, such as baseline proprioceptive acuity, vestibular function, and individual sensory integration strategies.

In light of these considerations, while the study was appropriately powered based on *a priori* calculations and met the assumptions of normality, variance homogeneity, and sphericity for repeated-measures ANOVA, some statistical limitations should be noted. First, although Bonferroni corrections were applied to control for Type I error in *post hoc* comparisons, such adjustments may also increase the risk of Type II errors, potentially obscuring smaller but meaningful effects. Second, the generalizability of the findings may be limited by the sample size and demographic homogeneity of the participants. Third, the use of parametric tests assumes ideal data distributions, and future studies may consider complementary non-parametric approaches or Bayesian inference to validate these results. A more comprehensive multivariate analysis may also help clarify the role of individual differences in SPV responses.

## Conclusions

We hypothesized that unilateral NMV would shift SPV toward the contralateral side of the stimulus; however, the findings revealed that NMV primarily reduced SPV variability without altering tilt direction. This suggests that cervical muscle proprioceptors play an important role in the integration of sensory information, particularly when there is a discrepancy between visual or head and trunk sensory information. The effects of unilateral NMV on posture highlight the crucial role of sensorimotor integration in maintaining body orientation in space. Unilateral NMV may enhance the sensitivity of body perception; however, the influence of SPV measurement conditions and sample size limitations on generalizability should be considered. Based on the results of this preliminary study, unilateral NMV could be a promising approach for patients with SPV abnormalities.

## Supplemental Information

10.7717/peerj.20579/supp-1 Supplemental Information 1 All raw data for analysis.

## Additional Information and Declarations

### Data Availability

The following information was supplied regarding data availability:

The raw measurements are available in the Supplemental File.
